# Magnetic Drug Targeting Reduces the Chemotherapeutic Burden on Circulating Leukocytes

**DOI:** 10.3390/ijms14047341

**Published:** 2013-04-02

**Authors:** Christina Janko, Stephan Dürr, Luis E. Munoz, Stefan Lyer, Ricardo Chaurio, Rainer Tietze, Sarah von Löhneysen, Christine Schorn, Martin Herrmann, Christoph Alexiou

**Affiliations:** 1 Department of Internal Medicine 3, Friedrich-Alexander-University Erlangen-Nuremberg, Ulmenweg 18, Erlangen 91054, Germany; E-Mails: christina.janko@uk-erlangen.de (C.J.); luis.munoz@uk-erlangen.de (L.E.M.); ricardo.chaurio@uk-erlangen.de (R.C.); sarah.vl@gmx.de (S.L.); christine.schorn@uk-erlangen.de (C.S.); martin.herrmann@uk-erlangen.de (M.H.); 2 Department of Otorhinolaryngology, Head and Neck Surgery, Section for Experimental Oncology and Nanomedicine (SEON), University Hospital Erlangen, Waldstrasse 1, Erlangen 91054, Germany; E-Mails: stefan.lyer@uk-erlangen.de (S.L.); rainer.tietze@uk-erlangen.de (R.T.); christoph.alexiou@uk-erlangen.de (C.A.)

**Keywords:** chemotherapy, iron oxide nanoparticles, mitoxantrone, magnetic drug targeting, immune system, leukocytes

## Abstract

Magnetic drug targeting (MDT) improves the integrity of healthy tissues and cells during treatment with cytotoxic drugs. An anticancer drug is bound to superparamagnetic iron oxide nanoparticles (SPION), injected into the vascular supply of the tumor and directed into the tumor by means of an external magnetic field. In this study, we investigated the impact of SPION, mitoxantrone (MTO) and SPION^MTO^ on cell viability *in vitro* and the nonspecific uptake of MTO into circulating leukocytes *in vivo*. MDT was compared with conventional chemotherapy. MTO uptake and the impact on cell viability were assessed by flow cytometry in a Jurkat cell culture. In order to analyze MTO loading of circulating leukocytes *in vivo*, we treated tumor-bearing rabbits with MDT and conventional chemotherapy. *In vitro* experiments showed a dose-dependent MTO uptake and reduction in the viability and proliferation of Jurkat cells. MTO and SPION^MTO^ showed similar cytotoxic activity. Non-loaded SPION did not have any effect on cell viability in the concentrations tested. Compared with systemic administration *in vivo*, MDT employing SPION^MTO^ significantly decreased the chemotherapeutic load in circulating leukocytes. We demonstrated that MDT spares the immune system in comparison with conventional chemotherapy.

## 1. Introduction

**Figure 1:**
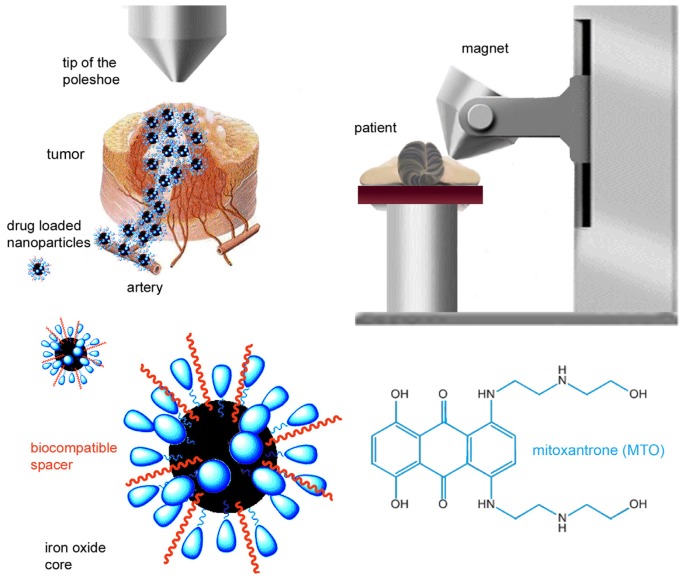
Principles of magnetic drug targeting. Superparamagnetic iron oxide nanoparticles (SPION) coated with lauric acid are loaded with mitoxantrone (SPION^MTO^). The solution is injected into the vascular supply of the tumor and accumulated at the intended site by means of an external magnetic field.

Together with surgery and radiotherapy, chemotherapy is still a mainstay of cancer treatment today. Its main disadvantage lies in the severe systemic adverse effects inherent in this mode of therapy. In order to kill tumor cells directly, anticancer drugs are usually given at the maximum dose tolerated by the patient [1], which means that the dose necessary for complete tumor eradication is barely achieved. Targeted drug delivery to the tumor may overcome this dilemma [2], and nanotechnology provides new possibilities in this rapidly growing medical field, e.g., in otorhinolaryngology [3]. Magnetic drug targeting (MDT), a new and innovative approach, has been developed to concentrate anticancer drugs in the tumor and reduce leakage into healthy tissues and cells. Superparamagnetic iron oxide nanoparticles (SPION) are functionalized with a chemotherapeutic agent, injected into the vascular supply of the tumor, and directed into the tumor itself by means of an external magnetic field (Figure 1) [4].

Systemic distribution and adverse drug reactions are thereby decreased, at the same time as the local impact on the tumor is increased. Lower doses of the anticancer drug can be administered, as enhancement rates are 26 times higher with MDT than with conventional systemic chemotherapy [5]. Previous animal studies with an experimental VX-2 squamous cell carcinoma in rabbits successfully demonstrated MDT-induced tumor remission using nanoparticle-coupled mitoxantrone (MTO) [6]. MTO acts against a variety of hematologic and solid tumors by impairing DNA replication, transcription and repair [7], and may cause adverse effects such as nausea, fever, anemia, immunosuppression, and cardiotoxicity [8].

The immune system plays a key role in carcinogenesis and cancer therapy. The initiation, growth and metastasis of a tumor require “cancer immunoediting” [9]. In addition, more and more attention is being paid to the immunological aspects of cancer therapy: antibodies [10], vaccines [11], and the induction of anti-tumor immunity during radiotherapy and chemotherapy [12]. This pilot study aimed to quantify MTO in peripheral blood leukocytes (PBL) using its inherent fluorescent properties, since PBL are crucial components of the immune system. We observed that targeted delivery of MTO by MDT reduces the cytostatic burden on circulating leukocytes.

## 2. Results and Discussion

### 2.1. Analysis of SPION by Flow Cytometry

**Figure 2:**
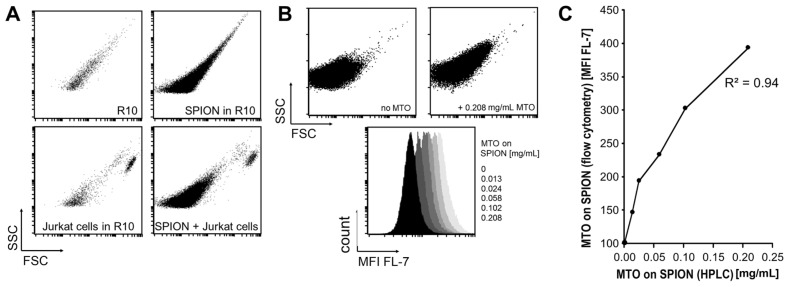
Analysis of superparamagnetic iron oxide nanoparticles (SPION) by flow cytometry. (**A**) Morphological analysis of SPION. Because of their small size, SPION can be clearly distinguished from Jurkat cells by forward and side scatter analysis (FSC/SSC) in flow cytometry. 0.5 × 10^6^ Jurkat cells in 500 μL R10 medium and 10 μL of SPION (5.7 μg/μL Fe) were analyzed at a low flow rate setting; (**B**) Mitoxantrone-loaded SPION (SPION^MTO^) show higher MFI [FL-7] values than non-loaded SPION. MTO coupling results in an increase of FSC and SSC (upper panel). The MFI of 10 μL pure SPION and 10 μL SPION loaded with various amounts of MTO in 500 μL phosphate buffered saline (PBS)/20% fetal calf serum (FCS) are shown, and clearly demonstrate that the MFI [FL-7] depends on the MTO content (lower panel). Measurements were performed at a medium flow rate setting; (**C**) Analysis of MTO loading on SPION by high performance liquid chromatography (HPLC) and flow cytometry. 10 μL SPION^MTO^ were diluted in 500 μL PBS/20% FCS and the measurement was performed at a medium flow rate setting. There was a good correlation between the quantification of MTO by HPLC and the flow cytometry of SPION^MTO^ (*R*^2^ = 0.94).

SPION can be detected by flow cytometry, as shown in Figure 2A. We found a clear distinction from Jurkat cells in size and granularity. Forward and side scatter analysis (FSC/SSC blots) showed SPION as a cloud of events two orders of magnitudes smaller and less granular than the cells. This difference allowed us to gate cells without contaminating SPION.

Since MTO is inherently fluorescent, it is possible to detect and quantify MTO bound to SPION (SPION^MTO^) by flow cytometry. The flow cytometer uses separate fluorescence channels to detect emitted light: MTO was excited with 638 nm and the fluorescent emission was recorded with a 725 ± 10 nm band pass detector in channel 7 [FL-7]. Binding MTO to SPION resulted in a moderate increase in the SPION FSC and SSC signals (Figure 2B, upper panel) and caused an MTO dose-dependent shift in the FL-7 signal when compared with non-loaded SPION (Figure 2B, lower panel).

High performance liquid chromatography (HPLC) analysis of detached MTO is an established method of analyzing drug loading efficiency on SPION. We found a clear correlation (*R*^2^ = 0.94) between concentrations of SPION^MTO^ calculated by HPLC and the mean fluorescence intensity (MFI) values determined by flow cytometry. Dilution of SPION^MTO^ decreased the number of events gated by FSC/SSC and reduced the MFI (Figure 2C).

### 2.2. Uptake of MTO and SPION^MTO^ by Jurkat Cells

**Figure 3:**
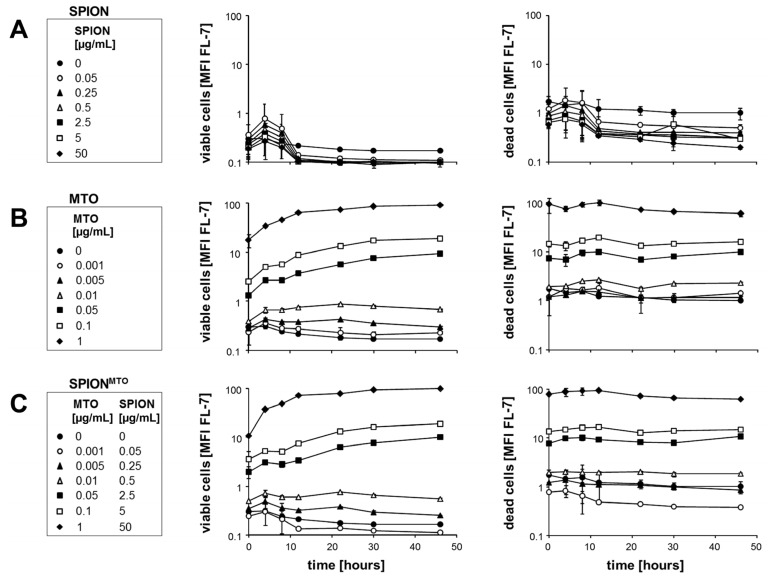
Uptake of MTO from superparamagnetic iron oxide nanoparticles (SPION), mitoxantrone (MTO), and MTO-loaded SPION (SPION^MTO^) by Jurkat cells. Cells were classed as viable or dead, depending on their morphological properties. (**A**) SPION-treated cells showed only autofluorescence; (**B**) Viable cells treated with MTO showed an increase in MFI [FL-7] with time, while no increase in MFI occurred in dead cells; (**C**) SPION^MTO^-treated cells behaved similarly to cells incubated with pure MTO. In all cases, the MFI [FL-7] was dependent on MTO concentrations. Figures show the mean values of duplicates with standard deviations.

To gain information about the *in vitro* uptake of MTO in cell culture, we treated Jurkat cells with MTO and SPION^MTO^ in various concentrations and used flow cytometry to examine viable and dead Jurkat cells (Figure 3). Cells were classified as viable or dead, according to their morphological properties. SPION-treated cells, both viable and dead, exhibited only low autofluorescence. When we incubated viable cells with various concentrations of pure MTO, we found a dose-dependent increase in MFI [FL-7]. Whereas dead cells displayed a flat MFI signal over time, fluorescence increased in viable cells for at least 24 h. Most importantly, there was no relevant difference between cells incubated with pure MTO and those with SPION^MTO^. The MFI signals were strongly dependent on the MTO concentration in both cases, demonstrating that MTO and SPION^MTO^ display similar cytotoxicity *in vitro*.

### 2.3. Proliferation of Jurkat Cells Treated with SPION, MTO and SPION^MTO^

**Figure 4:**
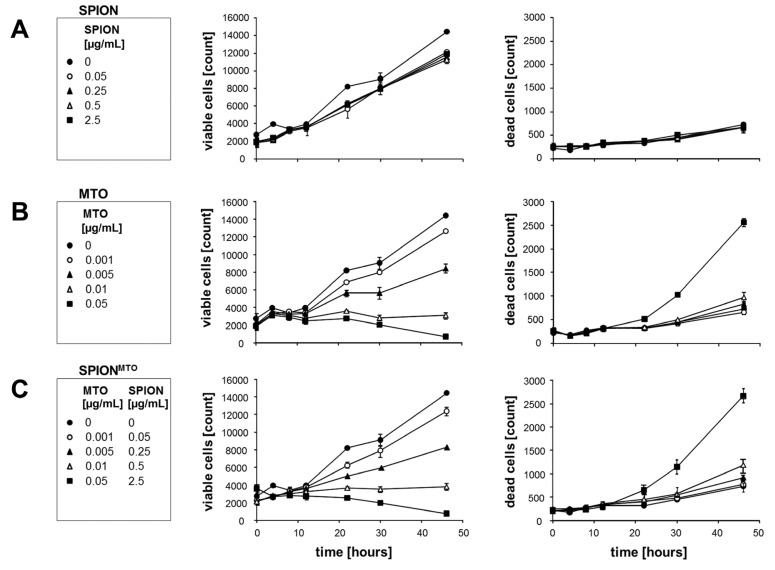
Proliferation of Jurkat cells treated with superparamagnetic iron oxide nanoparticles (SPION), mitoxantrone (MTO), and MTO-loaded SPION (SPION^MTO^). Viable and dead cells were distinguished according to their morphological properties (absolute values shown). (**A**) Pure SPION did not induce cell death in the tested concentrations; (**B**) Depending on the pure MTO concentration, cells showed reduced proliferation and an increased cell death rate; (**C**) SPION^MTO^ demonstrated similar behavior to MTO. Figures show the mean values of duplicates with standard deviations.

We then analyzed the proliferation rate of Jurkat cells treated with SPION, MTO and SPION^MTO^ using flow cytometry (Figure 4). Viable and dead cells were distinguished by their morphological properties (absolute values are shown).

Untreated cells show marked proliferation. Treatment with 0.001 μg/mL pure MTO or SPION^MTO^ affected cell proliferation slightly, while 0.005 μg/mL pure MTO or SPION^MTO^ had severe effects. MTO or SPION^MTO^ at a concentration of 0.01 μg/mL completely abolished proliferation. Incubation of Jurkat cells with pure MTO or SPION^MTO^ concentrations above 0.05 μg/mL resulted in cell death starting 24 h after treatment. In summary, MTO and SPION^MTO^ induced similar proliferation and cell death rates *in vitro*.

### 2.4. Apoptosis and Necrosis of Jurkat Cells Treated with SPION, MTO and SPION^MTO^

In viable cells, phosphatidylserine (PS) is found mainly in the inner leaflet of the plasma membrane. During apoptosis, PS is exposed on the cell surface. Redistribution of PS in apoptosis and the loss of plasma membrane integrity in necrosis were monitored by annexinV-FITC/propidium iodide (AxV/PI) staining.

**Figure 5:**
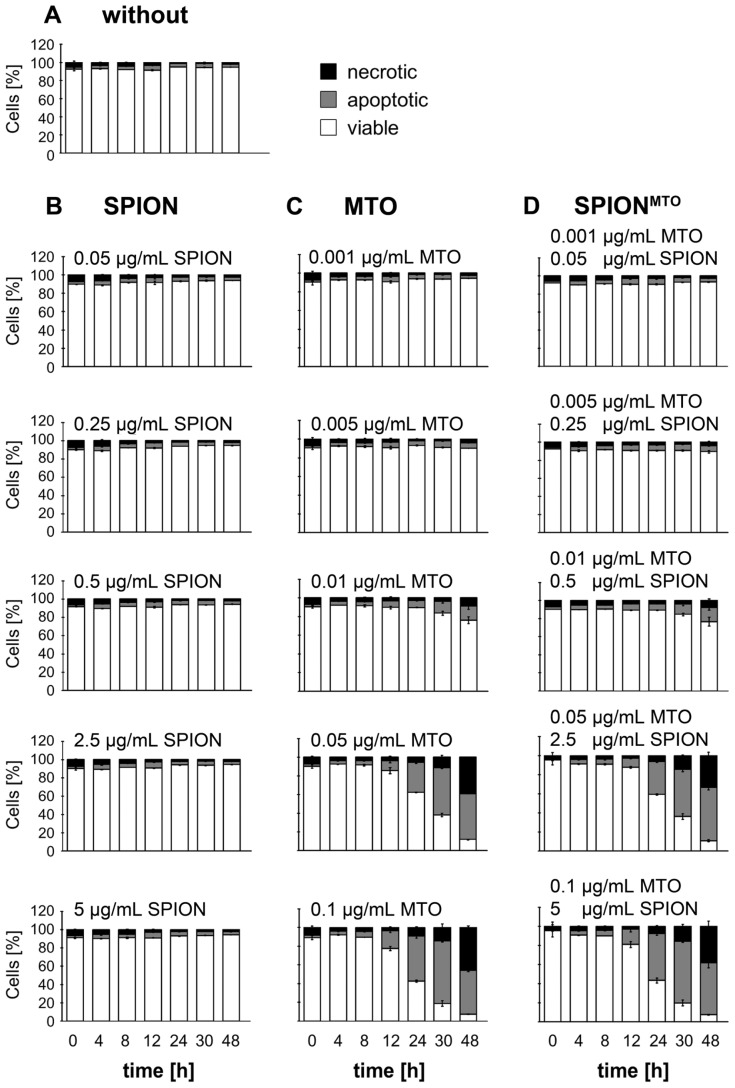
Cell death of Jurkat cells after treatment with superparamagnetic iron oxide nanoparticles (SPION), mitoxantrone (MTO), and MTO-loaded SPION (SPION^MTO^). Cell death was determined by annexinV-FITC/propidium iodide (AxV/PI) staining. AxV−/PI−, AxV+/PI−, and PI+ cells were considered viable, apoptotic and necrotic, respectively. Cells were incubated with various concentrations of SPION, MTO and SPION^MTO^ and then stained with AxV/PI at various points in time. (**A**) Mock-treated cells served as control; (**B**) Non-loaded SPION did not induce cell death, whereas (**C**) MTO and (**D**) SPION^MTO^ induced apoptosis in a dose-dependent manner, with secondary necrosis after prolonged incubation. Figures show the mean values of duplicates with standard deviations.

We incubated Jurkat cells with SPION, MTO and SPION^MTO^ in various concentrations for 48 h and analyzed the rate of viable (AxV−/PI−), apoptotic (AxV+/PI−) and necrotic cells (PI+) at several points in time (Figure 5). Mock-treated cells served as controls. Most untreated and SPION-treated cells maintained a viable phenotype throughout the experiment. Neither MTO nor SPION^MTO^ induced cell death in Jurkat cells at concentrations of MTO ≤ 0.01 μg/mL, whereas both induced apoptosis and secondary necrosis at concentrations of 0.05 μg/mL and above (Figure 5).

### 2.5. MDT Reduced the MTO Load of Circulating Leukocytes *in Vivo*

**Figure 6:**
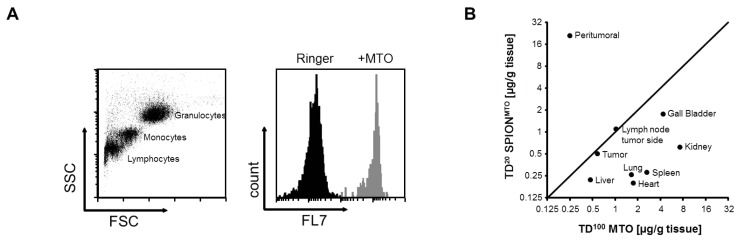

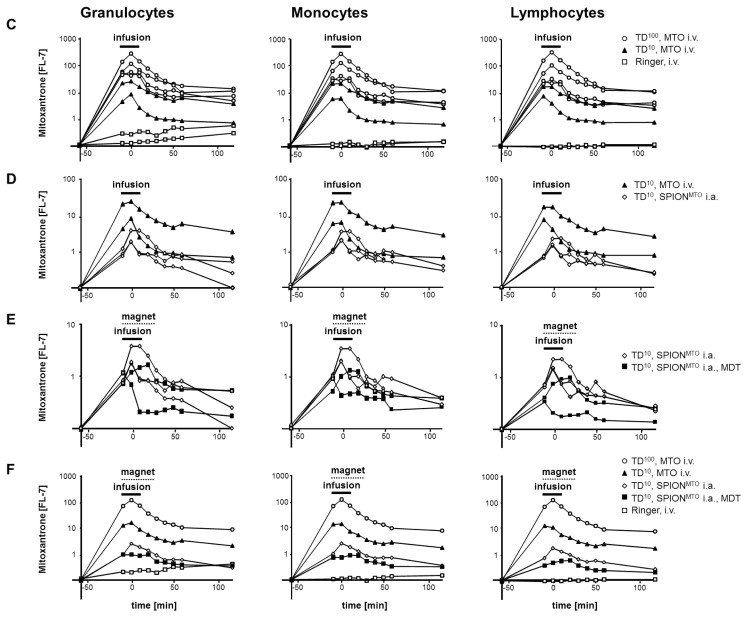
Magnetic drug targeting (MDT) prevents exposure of healthy circulating leukocytes to mitoxantrone (MTO). Blood was taken before (−60 min), during (−10 min, 0 min, +10 min), and after (+20 min, +30 min, +40 min, +50 min, +60 min, +120 min) administration of the drug. (**A**) Flow cytometry of rabbit blood. Before flow cytometry, erythrocytes were lysed by hypotonic treatment in rabbit whole blood; granulocytes, monocytes and lymphocytes were distinguished by their morphological features (FSC: size; SSC: granularity). MTO uptake into blood cells was monitored by flow cytometry employing MFI [FL-7]; (**B**) After MDT, MTO accumulates primarily in the tumor, with MTO concentrations in the central tumor and neighboring lymph nodes being similar. The load was reduced in all other organs; (**C**) MTO incorporated into circulating leukocytes depended on the systemic dose administered. TD^100^ MTO (*n* = 4), TD^10^ MTO (*n* = 2), and pure Ringer’s solution (*n* = 2) were injected intravenously; (**D**) Coupling MTO to superparamagnetic iron oxide nanoparticles (SPION) and injecting the resultant SPION^MTO^ into an artery supplying the tumor reduces the amount of MTO in circulating leukocytes. TD^10^ MTO (*n* = 2) was given intravenously and TD^10^ SPION^MTO^ (*n* = 3) injected intra-arterially; (**E**) Routing SPION^MTO^ into the tumor region with an external magnetic field further reduced circulating leukocyte exposure to MTO. TD^10^ SPION^MTO^ (*n* = 5) was administered directly into the arterial supply of the tumor. One group (*n* = 2) was exposed to an external magnetic field (MDT); (**F**) Data on MTO uptake show that MDT significantly reduced the MTO burden of circulating leukocytes: conventional chemotherapy with TD^100^ MTO i.v. (*n* = 4), TD^10^ MTO i.v. (*n* = 2), TD^10^ SPION^MTO^ i.a. (*n* = 3), TD^10^ SPION^MTO^ i.a. with exposure to an external magnetic field (*n* = 2), and controls treated with Ringer’s solution (*n* = 2).

Exposing healthy tissues and cells to anticancer drugs is a crucial problem with conventional chemotherapy and causes multiple adverse reactions (e.g., leukopenia). To study this effect, we investigated MTO contamination in PBL during conventional chemotherapy and MDT in an *in vivo* rabbit tumor model. Using flow cytometry, we distinguished granulocytes, monocytes, and lymphocytes by their morphological features and quantified the MTO content as MFI [FL-7] (Figure 6A). As shown previously [5], MDT with a 20% therapeutic dose (TD^20^) of SPION^MTO^ reduces the chemotherapeutic burden on healthy tissue and enhances MTO in the tumor (Table 1, Figure 6B).

We detected increasing MTO concentrations in all leukocytes immediately after starting the infusion. MTO concentrations reached their peak after 10 min. MTO loading started to decline as soon as the MTO infusion finished (after 20 min). Comparing conventional chemotherapy in rabbits given a 100% therapeutic dose (TD^100^) of MTO (*n* = 4), TD^10^ MTO (*n* = 2), or Ringer’s solution (*n* = 2) intravenously, we found that MTO uptake in PBL clearly depended on the systemic dose administered. Granulocytes, monocytes, and lymphocytes displayed similar MTO profiles (Figure 6C).

Next, we studied the uptake of SPION^MTO^ into PBL. We compared the MTO content of PBL after intravenous injection of TD^10^ MTO with that found after intra-arterial injection of TD^10^ SPION^MTO^. PBL had a reduced MTO load after SPION^MTO^, but the difference was not statistically significant (Figure 6D).

An important step in MDT is focusing SPION^MTO^ on the target area with an external magnetic field. After injecting TD^10^ SPION^MTO^ into an artery directly supplying the tumor, MDT was applied to one group of rabbits (MDT), while the second group was not exposed to the magnetic field. As shown in Figure 6E, MTO uptake by PBL decreased even further. Compared with conventional chemotherapy, MDT resulted in lower MTO loading of circulating PBL—A difference that is statistically significant (*p* = 0.014, paired Student’s *t*-test).

Figure 6F summarizes the mean MTO loads of PBL in the different groups of rabbits. We compared TD^100^ MTO i.v. (*n* = 4), TD^10^ MTO i.v. (*n* = 2), TD^10^ SPION^MTO^ i.a. (*n* = 3), TD^10^ SPION^MTO^ i.a. MDT (*n* = 2) and controls treated with Ringer’s solution (*n* = 2). We demonstrated that PBL take up MTO in a dose-dependent manner, and that coupling MTO to SPION decreases the (unwanted) MTO load in PBLs, especially when MDT is applied. In summary, MDT significantly reduced the MTO burden of healthy PBL in comparison with conventional chemotherapy.

## 3. Experimental Section

### 3.1. Tumor Model

An experimental VX-2 tumor was implanted subcutaneously into the left hind limb of female New Zealand white rabbits (Charles River Laboratories, Sulzfeld, Germany), aged 18–22 weeks and weighing 3.1–3.9 kg. After four to six weeks, the tumor had grown to a size that made intervention necessary.

### 3.2. Chemotherapeutic Agent

Mitoxantrone (Mitoxantrone NC 2 mg/mL; NeoCorp AG, Weilheim, Germany) is an anticancer drug of the anthracenedione class. MTO acts by intercalating (hydrogen bonds) into DNA, inducing crosslinks and strand breaks [13]; it also inhibits the enzyme topoisomerase II [14].

### 3.3. Nanoparticles

The superparamagnetic iron oxide nanoparticles (SPION), composed of maghemite (Fe_2_O_3_) and magnetite (Fe_3_O_4_), were produced by SEON. Alkaline precipitation was followed by self-assembling functionalization with lauric acid and MTO [15]. The hydrodynamic diameter was set at approximately 100 nm using dynamic light scattering. MTO was bound to SPION (SPION^MTO^) in an aqueous solution (ferrofluid). To analyze the MTO-content of the ferrofluid, MTO was extracted from 100 μL ferrofluid by the addition of 900 μL 1N HCl and incubation in a Thermomixer^®^ comfort (Eppendorf AG, Hamburg, Germany) at 600 rpm and room temperature for 1 h. After centrifuging with a Minispin^®^ plus (Eppendorf AG, Hamburg, Germany) at 14,500 rpm for 10 min, the supernatant was measured with HPLC, using a Waters Alliance model consisting of a separation module (2695 series), a dual wavelength absorbance detector (2487 series), and a 3.0 × 100 mm X-Bridge phenyl column (Waters, Germany). The mobile phase consisted of formiate buffer (pH 3.0) and methanol (80:20 *v*/*v*) [16].

### 3.4. Mode of Administration

Therapy was given as a single treatment cycle. We administered conventional chemotherapy intravenously into an ear vein, while MDT required intra-arterial access. For this purpose, the rabbits were taken to an animal operation theatre and the femoral artery prepared under sterile conditions. Pure MTO and drug-loaded SPIONs, as well as Ringer’s solution, were injected via a 24 G i.v. catheter (B. Braun, Melsungen, Germany) over 20 min. In one group, an electromagnet (Siemens Healthcare, Erlangen, Germany) with a magnetic field gradient of 72 T/m located directly beneath the pole tip [17] attracted the drug-loaded nanoparticles to the tumor during the 20 min of drug administration and for a further 15 min afterwards. Blood was taken before administration (−60 min), during the infusion (−10 min, 0 min, +10 min), and afterwards (+20 min, +30 min, +40 min, +50 min, +60 min, +120 min). For whole blood analyses, the erythrocytes in rabbit EDTA-blood were subjected to hypotonic lysis twice before flow cytometry.

### 3.5. Cells and Culture Conditions

To investigate the effects of SPION, MTO and SPION^MTO^ on mammalian cells, we used the non-adherent human T cell leukemia cell line Jurkat (ACC 282, DSMZ, Braunschweig, Germany). Cell culture was performed at 37 °C and 5.5% CO_2_ in R10 medium (RPMI 1640 medium supplemented with 10% FCS, 1% glutamine, 1% penicillin-streptomycin (all from Life Technologies GmbH, Darmstadt, Germany), and 1% HEPES (10 mM, pH 7.2) (Merck KGaA, Darmstadt, Germany)). Cells were counted and adjusted to a density of 0.5 × 10^6^ cells/mL in R10 medium for these experiments.

### 3.6. Measurement of Cellular Morphology

Dying cells change in morphology, and this can be detected by scatter alterations in flow cytometry [18]. Apoptosis is indicated by cell shrinkage, increased cytoplasmic granularity and vacuolization. Apoptotic cells are therefore detected as a population with decreased FSC and increased SSC [19].

### 3.7. Detection of Exposed Cell Phosphatidylserine and Analysis of Membrane Integrity

Recombinant chicken annexin V (AxV) (responsif GmbH, Erlangen, Germany) was labeled with fluorescein isothiocyanate (FITC) according to the manufacturer’s instructions (Sigma-Aldrich GmbH, Taufkirchen, Germany). AxV-FITC binds to exposed PS on the membranes of cells undergoing apoptosis. Before flow cytometry, 100 μL cell suspension was incubated with 400 μL freshly prepared staining solution, containing 1 μg/mL AxV-FITC and 20 μg/mL propidium iodide (PI) (Sigma-Aldrich GmbH, Taufkirchen, Germany) in Ringer’s solution, for 30 min at 4 °C. The PI was added to differentiate between apoptotic (AxV positive, PI negative), necrotic (AxV positive, PI positive) and viable (AxV negative, PI negative) cells [20].

### 3.8. Flow Cytometry

Flow cytometry was performed with a Gallios cytofluorometer™ (Beckman Coulter, Fullerton, CA, USA). The excitation wavelength for FITC and PI was 488 nm; FITC fluorescence was recorded by the FL-1 sensor (525/38 nm band pass), PI fluorescence by the FL-3 sensor (620/30 nm band pass). MTO fluorescence was excited at 638 nm and recorded by the FL-7 sensor (725/20 nm band pass). Electronic compensation was used to eliminate any fluorescence bleed-through. Data were analyzed with Kaluza™ software (Beckman Coulter, Fullerton, CA, USA).

## 4. Conclusions

MDT reduces the chemotherapeutic burden of circulating leukocytes. In our experiments, we used SPION^MTO^ with a high magnetic field gradient of 72 T/m, and compared this approach with conventional chemotherapy. As previously demonstrated using MDT (TD^20^ SPION^MTO^), we decreased the chemotherapeutic burden on healthy tissue and enhanced MTO in the tumor (Table 1, Figure 6B) [5].

Flow cytometry is the most appropriate technique for precise analysis at the single cell level. MTO and the closely related anthracycline antibiotics, daunorubicin, doxorubicin and idarubicin (data not shown) are clearly detectable by this method because of their inherent fluorescence [21]. Over the years, the technique has been improved [22]. In this study, we used flow cytometry to monitor the incorporation of MTO into non-adherent cells in cell cultures *in vitro* as well as into blood cells *in vivo*.

Using MFI [FL-7], it was also possible to determine the MTO loading efficiency of SPION. Although flow cytometry cannot detect a single SPION because of its small size, we established a measurement strategy to quantify MTO loading under defined conditions. Depending on the amount of MTO bound to the particles, SPION had a hydrodynamic diameter of approximately 100 nm, as determined by dynamic light scattering. Under defined conditions of SPION content, dilution, and flow rate, quantifying MTO bound to SPION in this way can compete with the established HPLC method used to determine loading efficiency [16].

Using flow cytometry, we found a dose-dependent effect of MTO and SPION^MTO^ on the proliferation and viability of non-adherent human Jurkat cells *in vitro*. The two compounds had very similar effects, confirming equal cytotoxic activity of these substances if they are not differentially routed by MDT. This finding confirmed earlier results of real-time cell analysis and lactate dehydrogenase release in adherent cancer cells [23]. As shown in previous studies, low and intermediate concentrations of non-loaded SPIONs did not affect cell viability [23]. In contrast to earlier experiments performed with MCF-7 cells (human breast carcinoma) [23], much lower MTO concentrations induced Jurkat cell death. This confirms the high lymphoblast susceptibility to MTO. AxV/PI staining identified MTO-induced cell death as early apoptosis or the later response of secondary necrosis.

Preservation of the immune system during cancer therapy is extremely important and the cytotoxic load in PBL can be used as a surrogate marker for the immune system burden. Unwanted cytotoxic drug uptake by PBL is a major obstacle in MTO dose escalation. Ballestrero therefore developed a treatment strategy compatible with the rescue of blood progenitor cells. He achieved an acceptable hematologic toxicity while escalating the dose of MTO up to 90 mg/m^2^[8].

During the past few decades, evidence emerged that low-dose chemotherapy has immunomodulatory or immunostimulatory effects [24]. Interestingly enough, it was the anthracyclines in particular which were reported to induce a specific anticancer immune response [25–27]. After the injection of mitoxantrone-treated B16-F1 cells into mice, their splenocytes displayed an increased cytolytic effect on B16-F1 cells [28].

Nanoparticles reportedly influence the immune system, causing either immunostimulation or immunosuppression [29]. CoCr nanoparticles, for example, have been found to inhibit T-cell proliferation, which may explain the fact that patients with metal-on-metal implants have reduced CD8^+^ T-cell populations [30]. Like chemotherapeutic agents, Fe_3_O_4_ (magnetite) magnetic nanoparticles, especially in low-doses, were found to affect immune function in mice [31]. In our *in vivo* and *in vitro* settings, SPION displayed no detectable cytotoxicity.

In comparison with conventional routes of administration, spillover of the chemotherapeutic agent MTO into healthy circulating PBL is significantly reduced by MDT-based treatment regimens. MDT reduces the major adverse effects of chemotherapy and preserves PBL, taken as a surrogate population of the immune system. We conclude that MDT saves drug and spares the immune system from cytotoxicity.

## Figures and Tables

**Table 1:** Distribution of MTO in organs (μg/g tissue) after conventional chemotherapy (TD^100^ MTO) and magnetic drug targeting (TD^20^ SPION^MTO^) [5].

	MTO content (μg/g tissue) after treatment	
	
Organ	Conventional chemotherapy (TD^100^ MTO)	Magnetic drug targeting (TD^20^ SPION^MTO^)
Tumor	0.58	0.50
Peritumoral	0.25	20.93
Ipsilateral lymph node	1.03	1.05
Heart	1.75	0.20
Lung	1.66	0.26
Liver	0.47	0.22
Gall bladder	4.38	1.75
Kidney	7.29	0.62
Spleen	2.65	0.28

## References

[1] Shimizu K, Oku N. (2004). Cancer anti-angiogenic therapy. Biol. Pharm. Bull..

[2] Tietze R, Lyer S, Durr S, Alexiou C. (2012). Nanoparticles for cancer therapy using magnetic forces. Nanomedicine (London, England).

[3] Durr S, Tietze R, Lyer S, Alexiou C. (2012). Nanomedicine in otorhinolaryngology—Future prospects. Laryngorhinootologie.

[4] Alexiou C, Tietze R, Schreiber E, Lyer S. (2010). Nanomedicine: Magnetic nanoparticles for drug delivery and hyperthermia—new chances for cancer therapy. Bundesgesundheitsblatt Gesundheitsforschung Gesundheitsschutz.

[5] Alexiou C, Jurgons R, Schmid R, Erhardt W, Parak F, Bergemann C, Iro H. (2005). Magnetic drug targeting—a new approach in locoregional tumor therapy with chemotherapeutic agents. Experimental animal studies. HNO.

[6] Alexiou C, Arnold W, Klein R.J, Parak F.G, Hulin P, Bergemann C, Erhardt W, Wagenpfeil S, Lubbe A.S. (2000). Locoregional cancer treatment with magnetic drug targeting. Cancer Res..

[7] Fox M.E, Smith P.J. (1990). Long-term inhibition of DNA synthesis and the persistence of trapped topoisomerase II complexes in determining the toxicity of the antitumor DNA intercalators mAMSA and mitoxantrone. Cancer Res..

[8] Ballestrero A, Ferrando F, Garuti A, Basta P, Gonella R, Esposito M, Vannozzi M.O, Sorice G, Friedman D, Puglisi M. (1997). High-dose mitoxantrone with peripheral blood progenitor cell rescue: Toxicity, pharmacokinetics and implications for dosage and schedule. Br. J. Cancer.

[9] Dunn G.P, Bruce A.T, Ikeda H, Old L.J, Schreiber R.D. (2002). Cancer immunoediting: From immunosurveillance to tumor escape. Nat. Immunol..

[10] Scott A.M, Wolchok J.D, Old L.J. (2012). Antibody therapy of cancer. Nat. Rev. Cancer.

[11] Palucka K, Ueno H, Banchereau J. (2011). Recent developments in cancer vaccines. J. Immunol..

[12] Frey B, Rubner Y, Wunderlich R, Weiss E.M, Pockley A.G, Fietkau R, Gaipl U.S. (2012). Induction of abscopal anti-tumor immunity and immunogenic tumor cell death by ionizing irradiation - implications for cancer therapies. Curr. Med. Chem..

[13] Kapuscinski J, Darzynkiewicz Z, Traganos F, Melamed M.R. (1981). Interactions of a new antitumor agent, 1,4-dihydroxy-5,8-bis[[2-[(2-hydroxyethyl)amino]-ethyl]amino]-9,10-anthracenedion e, with nucleic acids. Biochem. Pharmacol..

[14] Frank P, Novak R.F. (1985). Mitoxantrone and bisantrene inhibition of platelet aggregation and prostaglandin E2 production *in vitro*. Biochem. Pharmacol..

[15] Khalafalla S.E, Reimers G.W. (1980). Preparation of dilution-stable aqueous magnetic fluids. IEEE Trans. Magn..

[16] Tietze R, Schreiber E, Lyer S, Alexiou C (2010). Mitoxantrone loaded superparamagnetic nanoparticles for drug targeting: A versatile and sensitive method for quantification of drug enrichment in rabbit tissues using HPLC-UV. J. Biomed. Biotechnol..

[17] Alexiou C, Diehl D, Henninger P, Iro H, Rockelein R, Schmidt W, Weber H. (2006). A high field gradient magnet for magnetic drug targeting. IEEE Trans Appl. Supercon..

[18] Elstein K.H, Zucker R.M. (1994). Comparison of cellular and nuclear flow cytometric techniques for discriminating apoptotic subpopulations. Exp. Cell Res..

[19] Hagenhofer M, Germaier H, Hohenadl C, Rohwer P, Kalden J.R, Herrmann M. (1998). UV-B irradiated cell lines execute programmed cell death in various forms. Apoptosis.

[20] Vermes I, Haanen C, Steffens-Nakken H, Reutelingsperger C. (1995). A novel assay for apoptosis. Flow cytometric detection of phosphatidylserine expression on early apoptotic cells using fluorescein labelled Annexin V. J. Immunol. Methods.

[21] Smith P.J, Sykes H.R, Fox M.E, Furlong I.J. (1992). Subcellular distribution of the anticancer drug mitoxantrone in human and drug-resistant murine cells analyzed by flow cytometry and confocal microscopy and its relationship to the induction of DNA damage. Cancer Res..

[22] Chan A, Weilbach F.X, Toyka K.V, Gold R. (2005). Mitoxantrone induces cell death in peripheral blood leucocytes of multiple sclerosis patients. Clin. Exp. Immunol..

[23] Durr S, Lyer S, Mann J, Janko C, Tietze R, Schreiber E, Herrmann M, Alexiou C. (2012). Real-time cell analysis of human cancer cell lines after chemotherapy with functionalized magnetic nanoparticles. Anticancer Res..

[24] Apetoh L, Ghiringhelli F, Tesniere A, Obeid M, Ortiz C, Criollo A, Mignot G, Maiuri M.C, Ullrich E, Saulnier P. (2007). Toll-like receptor 4-dependent contribution of the immune system to anticancer chemotherapy and radiotherapy. Nat. Med..

[25] Zitvogel L, Apetoh L, Ghiringhelli F, Andre F, Tesniere A, Kroemer G. (2008). The anticancer immune response: Indispensable for therapeutic success?. J. Clin. Invest..

[26] Apetoh L, Mignot G, Panaretakis T, Kroemer G, Zitvogel L. (2008). Immunogenicity of anthracyclines: Moving towards more personalized medicine. Trends Mol. Med..

[27] Galluzzi L, Senovilla L, Zitvogel L, Kroemer G (2012). The secret ally: Immunostimulation by anticancer drugs. Nat. Rev. Drug Discov..

[28] Cao C, Han Y, Ren Y, Wang Y. (2009). Mitoxantrone-mediated apoptotic B16-F1 cells induce specific anti-tumor immune response. Cell Mol. Immunol..

[29] Zolnik B.S, Gonzalez-Fernandez A, Sadrieh N, Dobrovolskaia M.A. (2010). Nanoparticles and the immune system. Endocrinology.

[30] Ogunwale B, Schmidt-Ott A, Meek R.M, Brewer J.M. (2009). Investigating the immunologic effects of CoCr nanoparticles. Clin. Orthop. Relat. Res..

[31] Chen B.A, Jin N, Wang J, Ding J, Gao C, Cheng J, Xia G, Gao F, Zhou Y, Chen Y. (2010). The effect of magnetic nanoparticles of Fe_3_O_4_ on immune function in normal ICR mice. Int. J. Nanomed..

